# COVID-19 vaccine related hypermetabolic lymph nodes on PET/CT: Implications of inflammatory findings in cancer imaging

**DOI:** 10.32604/or.2023.027705

**Published:** 2023-04-10

**Authors:** FERDINANDO CALABRIA, ANTONIO BAGNATO, GIULIANA GUADAGNINO, MARIA TOTEDA, ANTONIO LANZILLOTTA, STEFANIA CARDEI, ROSANNA TAVOLARO, MARIO LEPORACE

**Affiliations:** 1 Department of Nuclear Medicine and Theragnostics, Mariano Santo Hospital, Cosenza, 87100, Italy; 2 Department of Infectious and Tropical Diseases, St. Annunziata Hospital, Cosenza, 87100, Italy

**Keywords:** [^18^F]Choline, 2-[^18^F]FDG, PET/CT, COVID-19, Vaccine, Italy, Chemotherapy, Inflammation, SUVmax

## Abstract

We observed several patients presenting 2-[^18^F]FDG uptake in the reactive axillary lymph node at PET/CT imaging, ipsilateral to the site of the COVID-19 vaccine injection. Analog finding was documented at [^18^F]Choline PET/CT. The aim of our study was to describe this source of false positive cases. All patients examined by PET/CT were included in the study. Data concerning patient anamnesis, laterality, and time interval from recent COVID-19 vaccination were recorded. SUVmax was measured in all lymph nodes expressing tracer uptake after vaccination. Among 712 PET/CT scans with 2-[^18^F]FDG, 104 were submitted to vaccination; 89/104 patients (85%) presented axillary and/or deltoid tracer uptake, related to recent COVID-19 vaccine administration (median from injection: 11 days). The mean SUVmax of these findings was 2.1 (range 1.6–3.3). Among 89 patients with false positive axillary uptake, 36 subjects had received chemotherapy due to lymph node metastases from somatic cancer or lymphomas, prior to the scan: 6/36 patients with lymph node metastases showed no response to therapy or progression disease. The mean SUVmax value of lymph nodal localizations of somatic cancers/lymphomas after chemotherapy was 7.8. Only 1/31 prostate cancer patients examined by [^18^F]Choline PET/CT showed post-vaccine axillary lymph node uptake. These findings were not recorded at PET/CT scans with [^18^F]-6-FDOPA, [^68^Ga]Ga-DOTATOC, and [^18^F]-fluoride. Following COVID-19 mass vaccination, a significant percentage of patients examined by 2-[^18^F]FDG PET/CT presents axillary, reactive lymph node uptake. Anamnesis, low-dose CT, and ultrasonography facilitated correct diagnosis. Semi-quantitative assessment supported the visual analysis of PET/CT data; SUVmax values of metastatic lymph nodes were considerably higher than post-vaccine lymph nodes. [^18^F]Choline uptake in reactive lymph node after vaccination was confirmed. After the COVID-19 pandemic, nuclear physicians need to take these potential false positive cases into account in daily clinical practice.

## Introduction

The first outbreak of *coronavirus disease-2019* (COVID-19) occurred during the early months of 2020; by the beginning of March, the virus had spread to all regions of Italy.

Worldwide, nuclear physicians described interstitial pneumonia with high uptake of 2-[^18^F]fluoro-2-deoxy-D-glucose (2-[^18^F]FDG) in PET/CT scans of asymptomatic cancer patients [1–3]. Anyway, 2-[^18^F]FDG PET/CT is not useful to integrate high-resolution CT in diagnosing COVID-19 infection [4].

Beyond the necessity of an accurate prophylaxis in nuclear medicine centers, the possibility to detect pulmonary uptake due to COVID-19 related pneumonitis is not the only feature to consider in clinical practice.

After the beginning of the Italian nationwide mass vaccination (for healthcare workers and oncologic patients) [5] we observed a significant amount of 2-[^18^F]FDG-avid axillary lymphadenopathies in oncologic patients examined by means of PET/CT, ipsilateral to the COVID-19 vaccine injection site. 2-[^18^F]FDG is a non-specific tumor marker and may be enhanced in inflammation.

Recent studies from Israel [6] and USA [7] already highlighted the identification of vaccine-related lymphadenopathies with moderate 2-[^18^F]FDG uptake in axillary regions after COVID-19 vaccine, following intramuscular administration in ipsilateral deltoid muscle.

Lymph node 2-[^18^F]FDG uptake in PET/CT was described after vaccines administration for human papillomaviruses and other vaccines [8]. Similar results were recorded after vaccination campaign against H1N1, in the site of injection (*deltoid muscle*) or in the ipsilateral axillary lymph nodes [6].

The fact that vaccines produce lymphatic activation is of major clinical significance. This feature may lead misdiagnoses in patients with lymphoma or breast carcinoma who had recent immunization. Tracer uptake in axillary lymph nodes can be falsely assumed to be due to malignant diseases, with negative effects on patients staging and therapy management.

Moreover, higher rates of 2-[^18^F]FDG uptake in reactive lymph nodes have been observed after administration of the available COVID-19 mRNA vaccines [9–11]. On the other hand, lymph node uptake due to inflammation is at the basis of a large amount of false positive cases occurring in patients examined by PET/CT with 2-[^18^F]FDG [12], [^18^F]Choline [13], and [^18^F]-6-FDOPA [14].

In our experience, from the beginning of the Italian national vaccination campaign, we progressively observed a large number of such findings in cancer patients examined by 2-[^18^F]FDG PET/CT. Moreover, we also observed similar features in a single prostate cancer patient examined by [^18^F]Choline PET/CT.

The aim of the study was to describe these cases observed in patients after mRNA based COVID-19 vaccination and to discuss peculiar findings observed at correlative imaging.

## Materials and Methods

From *January* to *April* 2021 we collected consecutive PET/CT findings of COVID-19 vaccine related tracer uptake.

### Data collection

In this time interval, we performed whole body PET/CT scans as follows: 712 patients with 2-[^18^F]FDG, 31 prostate cancer patients with [^18^F]Choline, 2 oncologic patients with [^18^F]-fluoride, 8 patients with neuroendocrine tumors by [^68^Ga]Ga-DOTATOC, 4 patients with medullary thyroid cancer with [^18^F]-6-FDOPA.

The study was approved by our local ethics committee. The need for informed patient consent was waived.

Nine patients undergoing whole body 2-[^18^F]FDG PET/CT scan were excluded due to incomplete medical records or a known malignancy involving axillary lymph nodes.

Before PET/CT, all patients were asked to fill a standard clinical form, including age, sex, clinical indication for the exam, clinical status, recent chemotherapy and/or radiotherapy, surgical procedures, date of COVID-19 vaccination and arm of vaccine administration.

When observed, correct identification of abnormal findings in axial and/or deltoid muscle were obtained by accurate anamnesis or, when possible, by correlative imaging with contrast enhanced CT and ultrasound, performed in our Department.

### PET/CT acquisition protocols

All patients fasted for at least 6 h before 2-[^18^F]FDG and [^18^F]Choline intravenous injection; in patients examined by 2-[^18^F]FDG PET/CT, serum glucose level was measured before tracer administration. Patients were injected with 250–330 MBq of 2-[^18^F]FDG and subsequently hydrated [*500 ml of intra venous saline sodium chloride (NaCl) 0.9%*] in order to reduce renal pooling of radiotracer.

A PET-CT system Discovery 710 (GE Medical Systems, TN, USA) was used. This system combines a high-speed ultra 128 slices CT unit and a PET scanner with 10080 bismuth germanate (BGO) crystals in 24 rings. A low amperage CT scan was acquired for attenuation correction of PET images (80 mA, 140 kV, field of view (FOV) about 420–500 mm, CT slice thickness 3.75). After low dose CT, PET images were acquired 60 min after 2-[^18^F]FDG administration (or 45 min after [^18^F]Choline administration).

### Imaging analysis

Images were evaluated on a dedicated workstation by a nuclear physician and a radiologist.

Axillary lymph nodes with tracer uptake, when observed during a PET/CT with 2-[^18^F]FDG, were evaluated by means of semi-quantitative analysis with maximum *Standardized Uptake Value* (SUVmax) [13]. Spherical Regions of Interest (ROIs) were drawn on fused axial PET/CT views to measure the SUVmax.

## Results

Globally, 757 consecutive adult patients were scanned by means of PET/CT in cited time interval.

### 2-[^18^F]FDG PET/CT

Particularly, we performed 712 2-[^18^F]FDG PET/CT scans in 357 male and 355 female patients (see Table 1).

**Table 1:** Demographic characteristics of whole examined population by means of PET/CT with several tracers. A minority of patients was submitted to COVID-19 vaccine

Tracer	Patients	Age	Male	Female	Vaccine
2-[^18^F]FDG	712	63 ± 9	357 (51%)	355 (49%)	104/712
[^18^F]Choline	31	65 ± 7	31	/	9/31
[^68^Ga]Ga-DOTATOC	8	59 ± 5	2	6	3/8
[^18^F]-6-FDOPA	4	58 ± 6	1	3	3/4
[^18^F]-fluoride	2	63 ± 1	2	/	1/2

Among these patients, 104/712 already received COVID-19 vaccine administration, with the first dose (n.63) or second dose (n.41). Thus, in our series, 653 patients undergoing 2-[^18^F]FDG PET/CT did not receive vaccine at the time of the scan.

**Figure 1:**
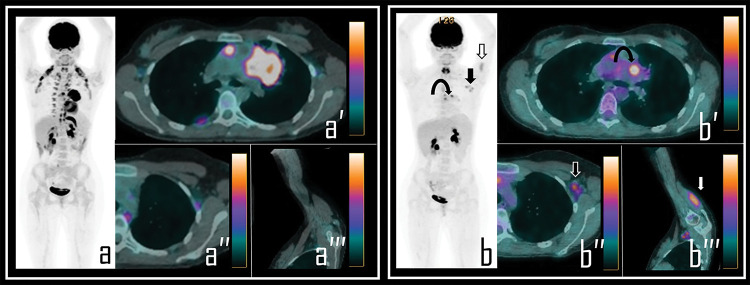
A 36-year-old male patient examined by 2-[^18^F]FDG PET/CT during the staging of *Hodgkin’s lymphoma*. Baseline PET/CT (a) showed a mediastinal *bulky* lymphoma, more evident in axial PET/CT view (a′). No abnormal foci of uptake were observed in left axilla neither in ipsilateral deltoid muscle, as showed in axial PET/CT detail (a′′) and oblique PET/CT view of the left arm (a′′′). Note: After 2 cycles of chemotherapy, the patient underwent *interim* 2-[^18^F]FDG PET/CT, showing incomplete response to therapy in the mediastinal mass with residual lymphoid active tissue, as evident in PET *Maximum Intensity Projection* (b, *curved arrow*). PET also showed focal uptake in the left deltoid (b, *white arrow*) and in three ipsilateral axillary lymph nodes (b, *black arrow*). Residual lymphoma is evident in axial PET/CT view (b′, *curved arrow*) while axial PET/CT detail shows a 2-[^18^F]FDG-avid lymph node in left axilla (b″, *black arrow*), with SUVmax 2.5; oblique PET/CT view of the left arm displays diffuse uptake in the deltoid muscle (b′′′, *white arrow*), with SUVmax 2.8. The Patient underwent first dose of COVID-19 vaccine 9 days before the second PET/CT scan.

**Figure 2:**
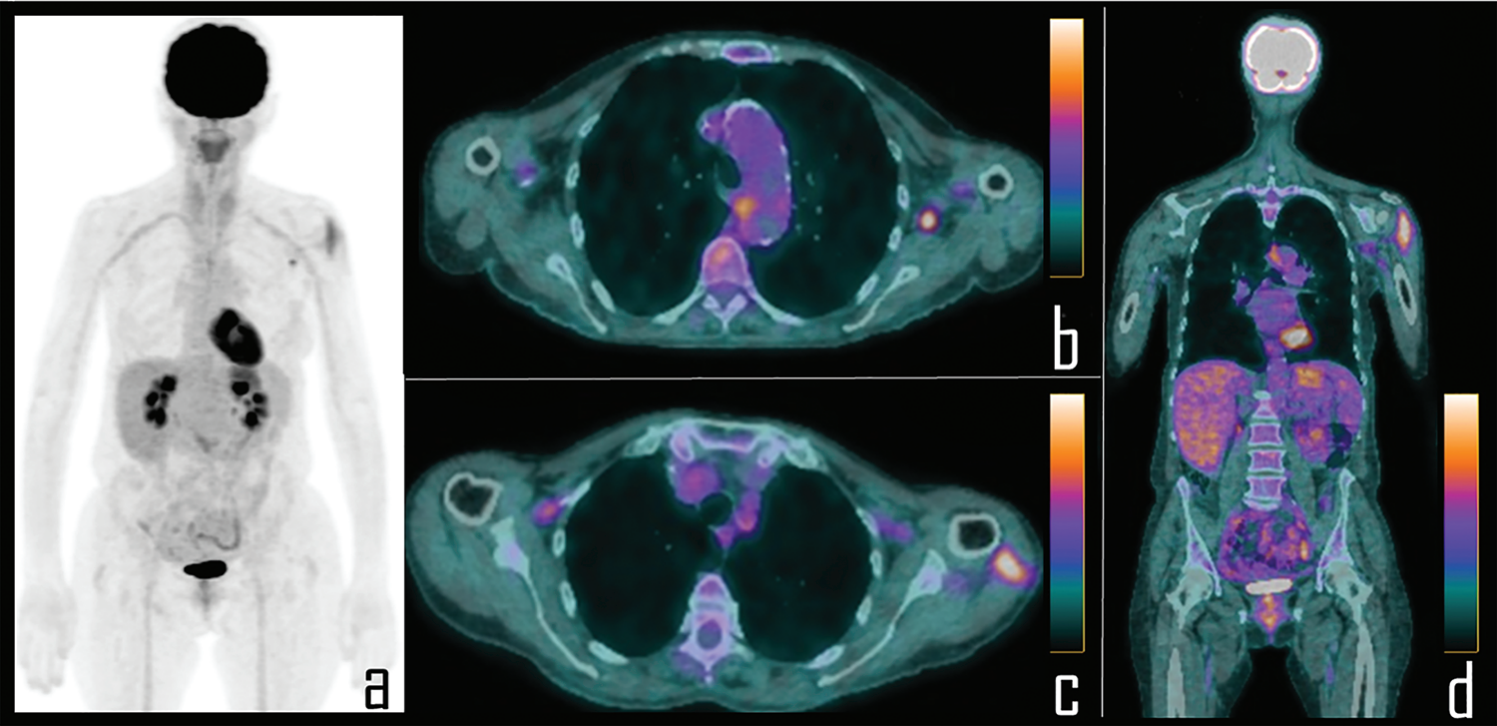
A 56-year-old female patient underwent 2-[^18^F]FDG PET/CT during follow up of colon cancer. PET *Maximum Intensity Projection* (a) was negative for disease relapse but showed focal area of uptake in the left axilla and diffuse tracer uptake in the ipsilateral arm. Axial PET/CT views better display the uptake respectively associated to a single axillary lymph node (b) and the ipsilateral deltoid muscle (c), which was the site of the second COVID-19 vaccine administration, occurred 8 days before the scan. Coronal PET/CT view (d) better displays diffuse uptake in the left deltoid muscle.

We observed axillary and/or deltoid 2-[^18^F]FDG uptake, related to COVID-19 vaccine administration, in 89/104 subjects (85%). Among these, 57/89 (64%) had received the first dose of vaccine prior to the PET/CT scan (Fig. 1) while 32/89 (36%) were also administered with the second dose (Fig. 2) of mRNA vaccine (see Table 2).

**Table 2:** Patients examined by 2-[^18^F]FDG PET/CT after the vaccine

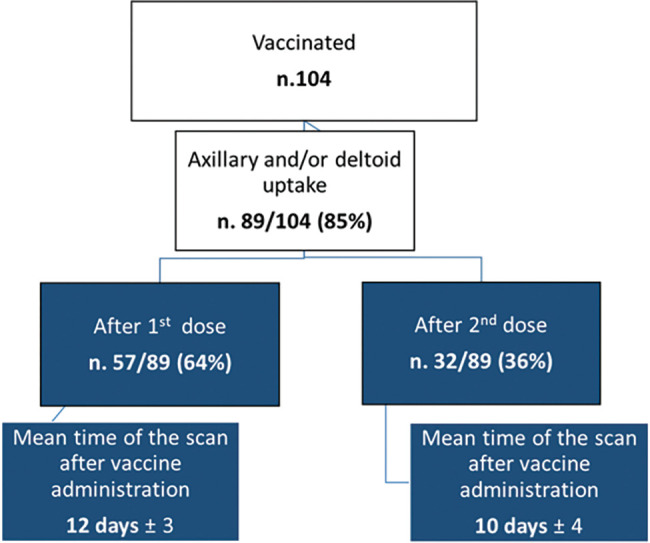

**Figure 3:**
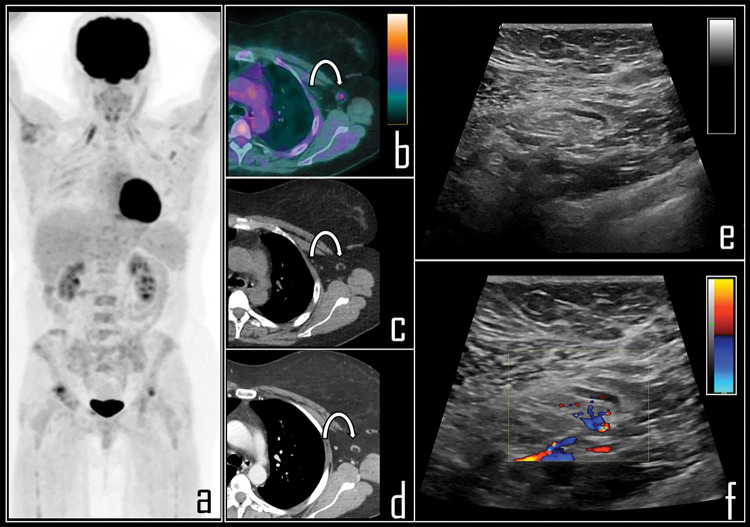
A 65-year-old female patient was examined by means of 2-[^18^F]FDG PET/CT during follow-up of ovarian cancer. PET *Maximum Intensity Projection* (a) was negative for disease relapse but showed a focal area of tracer uptake in the left axilla. Patient was previously submitted (14 days before) to second dose of COVID-19 vaccine in the deltoid muscle of left arm. Axial PET/CT view (b, *curved arrow*) shows the uptake in association with a 0.8 cm wide lymph node, with fatty hilum at correlative *low dose* axial CT (c, *curved arrow*). Subsequently, patient underwent whole body contrast enhanced CT, not showing disease relapse but confirming the axillary lymph node in left axilla, with low contrast enhancement (d, *curved arrow*). Ultrasonography was performed, displaying benign, 0.8 cm wide lymph node with regular shape (e), dominant central hilar flow and peripheral flow at color-doppler (f).

Mean time of the PET/CT scan after vaccine administration was 12 days (± 3) for patients submitted to the first vaccine dose and 10 days (± 4) for those patients who had also received the second dose (Fig. 3).

**Figure 4:**
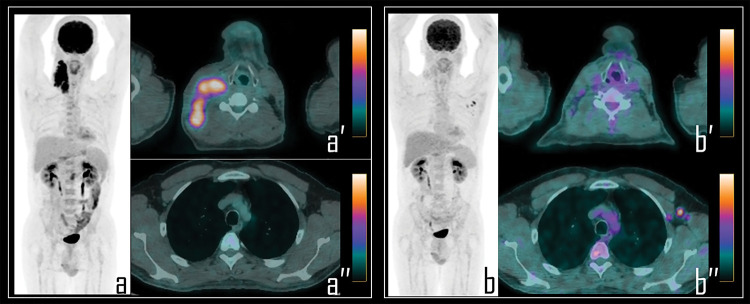
A 25-year-old male patient underwent 2-[^18^F]FDG PET/CT during the staging of *Hodgkin’s lymphoma*. PET (a) demonstrated pathologic tracer uptake in right lateral cervical lymphadenopathies. Axial PET/CT view well displays pathologic lymphadenopathies in the neck (a′) while no foci of uptake were detected in the axillary regions (a″). Subsequently, patient was submitted to chemotherapy and, 9 days before the interim PET/CT, to COVID-19 vaccine administration in the left arm. Note: Interim PET/CT (b) showed response to therapy with reduction of the uptake in the neck, more evident in axial PET/CT view (b″); conversely, three lymph nodes with moderate 2-[^18^F]FDG uptake were detected in the left axilla, as displayed in axial PET/CT view (b″), presenting SUVmax 2.8. These findings were linked to the previous vaccine administration.

Among 104 patients with vaccine-related 2-[^18^F]FDG false positive axillary findings, 36 patients (34%) received intravenous chemotherapy administration for lymph nodes metastases from somatic cancers or lymphomas, in the 15 days preceding the PET/CT scans (n.8 lymphomas, n.7 colon cancer, n.1 mesothelioma, n.12 ovarian cancers, n.8 lung carcinoma). In this subgroup of patients, 30 subjects presented complete response to therapy (Fig. 4) and 4 patients showed persistent disease; in 2/36 patients, lymph nodal progression disease was documented with newly diagnosed, infra-diaphragmatic, lymph node metastases.

Globally, reactive lymph node uptake in axillary lymph nodes and/or deltoid muscle was observed with a median from injection of 11 days.

Mean SUVmax values of lymph nodal localizations of somatic cancers/lymphomas was 7.8 (range 3.6–18.1).

Mean SUVmax recorded in axillary 2-[^18^F]FDG-avid lymph nodes after COVID-19 vaccine was 2.1 (range 1.6–3.3).

### [^18^F]Choline PET/CT and other tracers

**Figure 5:**
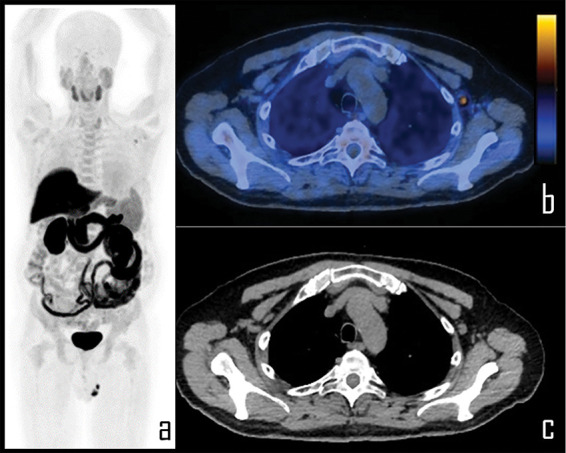
A 65-year-old prostate cancer patient underwent [^18^F]Choline PET/CT due to biochemical relapse of disease, two years after radical prostatectomy. PSA at the time of the scan was 0.4 ng/mL. Patient also underwent COVID-19 vaccine injection in the left arm 6 days before the exam. Note: PET imaging (a) was negative for disease relapse but showed a single area of focal [^18^F]Choline uptake in the left axilla, corresponding to a 1 cm wide, ovoid lymph node, displayed in axial PET/CT view (b), with fatty hilum detectable in the axial, co-registered *low dose* CT (c). This lymph node was considered as reactive, following vaccine administration; SUVmax was 2.3.

A single case of COVID-19 related tracer uptake in axillary lymph node was also documented during a whole body [^18^F]Choline PET/CT, among 31 prostate cancer patients examined with this tracer. Registered SUVmax in this lymph node was 2.3 (Fig. 5).

No similar findings were documented with other tracers routinely employed in our PET center, as [^18^F]-fluoride (n.2 patients), [^68^Ga]Ga**-**DOTATOC (n.8 patients) and [^18^F]-6-FDOPA (n.4 patients), despite vaccine was also administered in a minority of these selected patients (see Table 1).

## Discussion

Nuclear physicians need to be aware of the possibility to detect reactive, axillary, 2-[^18^F]FDG-avid lymph nodes during a PET/CT scan performed in cancer patients. In our series, 85% of examined population by 2-[^18^F]FDG PET/CT after COVID-19 mRNA vaccine administration, showed tracer-avid axillary lymph nodes, ipsilateral to the site of vaccine administration. No correlation was observed between number of doses of vaccine received by patients and node visualization.

### The role of hybrid and multimodal imaging

Hybrid nuclear medicine scans frequently permit to detect metabolic abnormalities without corresponding morphological changes; in our population, the detection of 2-[^18^F]FDG uptake in the muscular site of COVID-19 vaccine injection, associated to an ipsilateral tracer-avid lymph node, enabled recognition of the immune reaction following the vaccine.

Particularly, *low dose* CT of PET/CT was significantly important, allowing to correctly depict benign features of 2-[^18^F]FDG-avid lymph nodes. These *reactive* lymph nodes generally presented low size, regular margins, ovoid shape and fatty hilum [15,16]. In selected cases, the radiologist also performed extemporaneous ultrasonography of the axillary region immediately following PET/CT scan, aimed to ensure diagnosis.

Ultrasonography confirmed the presence of oval, hypoechoic and homogeneous lymph nodes, with evident fatty hilum, showing central vascularity at Color Doppler. All these features were suggestive of benign, reactive lymph nodes.

In malignant tumors potentially involving axillary and supraclavicular lymph nodes (breast cancer, lung carcinoma and lymphoma), the detection of tracer-avid lymph nodes due to vaccination may represent a diagnostic dilemma, as already documented also in melanoma patients [17]. Reasonably, we rather should consider the good benefit of ultrasonography on care of these patients.

### Semi-quantitative assessment in 2-[^18^F]FDG PET/CT patients

SUVmax measurement was confirmed as a valid semi-quantitative approach to improve visual analysis of PET data. All 2-[^18^F]FDG-avid reactive lymph nodes presented a SUVmax lower than 3.3, a value significantly concordant with inflammation [18].

In cancer patients examined by PET/CT, the high rate of 2-[^18^F]FDG uptake in benign lymph nodes is already known [12]: the accurate knowledge of CT and the availability of an ultrasound scanner in a nuclear medicine center can be of the utmost importance in correct identification of this source of false positive cases, minimizing misdiagnoses. This *translational* approach should be preferred by nuclear physicians, in order to perform more exhaustive reports.

However, among a large number of performed 2-[^18^F]FDG PET/CT scans, only 104 patients already received COVID-19 vaccine. This limit could be due to the choose time interval of the study, carried out immediately after the start of the national vaccination campaign. Moreover, we did not evaluate potential differences between the several kinds of COVID-19 mRNA vaccines used in Italy.

We also documented pathologic lymph nodal 2-[^18^F]FDG uptake in 36/89 patients undergoing chemotherapy for lymph node metastases from somatic cancers or lymphomas, presenting concomitant false positive axillary findings.

It is known that 2-[^18^F]FDG is a marker of tumor vitality and may detect metabolic modifications before morphologic lesions; in fact, functional PET imaging allows the evaluation of tumor metabolic response after chemotherapy earlier than morphologic imaging [19].

Some authors support the necessity of an 2-[^18^F]FDG PET/CT 15 days following chemotherapy, in order to assess tumor response [20], since the metabolic activity of the tumor significantly decreases, especially in comparison with a baseline PET/CT scan. The detection of reactive, tracer-avid lymph nodes in these patients can represent a diagnostic dilemma. In our series, we excluded patients with known malignancy involving axillary lymph nodes.

On the other hand, newly observed 2-[^18^F]FDG false positive axillary findings at PET imaging can mimic localizations of disease in patients with lymphomas. In particular, we observed axillary lymph node uptake in a patient with cervical *Hodgkin’s lymphoma* after two cycles of Bleomycin-Dacarbazine-Doxorubicin-Vinblastine (ABVD) chemotherapy protocol (Fig. 4); the interim PET/CT showed complete response to therapy while the reactive axillary findings were easily recorded as false positive, due to the documented metabolic response and the anamnesis. The visual, qualitative assessment of *interim* PET enabled correct diagnosis; comparison with baseline PET/CT scan was fundamental. In this field, the role of this diagnostic tool is ascertained, being PET/CT accurate predictor of patient overall survival, also leading change in chemotherapy when necessary, with reduced treatment-related toxic effects and total costs [21].

It is also evident that 2-[^18^F]FDG-avid axillary, reactive lymph nodes can overestimate the stage of disease, in patients with lymph nodal disease progression after chemotherapy.

On this topic, we registered a significant difference between metabolic activity expressed by tumor lesions and reactive lymph nodes. The SUVmax value was significantly higher in patients with stable disease or progression than in subjects with documented benign, inflammatory lymph nodes following vaccine administration. Moreover, chemotherapy seems not to influence this source of false positive, inflammatory findings [22].

Therefore, identification of 2-[^18^F]FDG uptake in deltoid and/or axillary lymph nodes, ipsilateral to recent intramuscular vaccine administration as not related to the underlying disease, can be facilitated by semi-quantitative assessment by SUVmax. Moreover, CT radiologic criteria of malignancy enable identification of pathologic lymphadenopathies expressing high glucose metabolism: *ill-defined borders, increased size, and nodal shape*.

Two patients showing disease progression after chemotherapy presented infra-diaphragmatic lymph nodes metastases; in these selected cases, recognition of such lesions was enabled respectively by localizations and glycolytic activity, higher than concomitant reactive axillary lymph nodes.

### Considerations and perspectives: a pragmatic approach

Our population is limited and not useful for a meaningful statistical analysis. Nevertheless, similarly to the work of other groups on this topic [4,23] our experience suggests some considerations.

After COVID-19 pandemic, nuclear physicians and radiologists need to be aware of vaccinated patients, due to potential high rate of false positive cases related to the vaccine, already reported in oncologic patients [24] examined at breast imaging [25], Magnetic Resonance Imaging (MRI) [26] and in PET/CT scans performed with 2-[^18^F]FDG [4], [^18^F]Choline [27] and somatostatin receptors analogs [28].

A practical management plan for radiologists and nuclear physicians, across specialties, has been already proposed, based on three key factors:

- timing and location of the vaccine injection;

- clinical context;

- imaging findings.

Referring to the clinical context, awareness of topographic distribution of lymph node metastases in metastatic cancer patients undergoing chemotherapy can be of help in differentiation between known lesions and new localizations from inflammatory findings [29].

Following suggestions by Lehman et al. [9], unilateral axillary lymphadenopathy, ipsilateral to recent vaccination, can be considered as benign and no further imaging is indicated. On the other hand, clinical management is recommended, with possibility of ultrasound if clinical concern or 2-[^18^F]FDG uptake persist two months after the final vaccination dose [6,30].

Concerning time interval of PET/CT after vaccine administration, we observed vaccine related 2-[^18^F]FDG uptake in axillary lymph nodes about 11 days after the vaccine injection, similarly to analogue study in breast cancer patients [30]; therefore, if the PET/CT is required in an urgent manner, as for staging cancer, no precautions are needed prior to the scan. In case of follow-up or restaging after chemotherapy, 2-[^18^F]FDG PET/CT may be delayed up to 15 days, trying to avoid false positive findings. Knowledge of date and site of vaccine administration, during data collection, is relevant to help nuclear physicians in promptly identifying vaccination as a potential cause of abnormal 2-[^18^F]FDG uptake. Thus, we modified anamnesis interviews preceding PET/CT [11].

Of course, oncologists may play an exclusive role in identification of those patients with known lymph node metastases, in which PET/CT scans may be slightly procrastinated. Vaccine administration in the arm contralateral to a unilateral cancer is preferable to avoid misdiagnosis.

Future studies on larger populations are needed to ensure the possibility of 2-[^18^F]FDG uptake in reactive axillary lymph nodes after vaccine administration and how long these findings can be observed.

### [^18^F]Choline, [^18^F]-6-FDOPA, [^68^Ga]Ga-DOTATOC, and [^18^F]-fluoride

The possibility of [^18^F]Choline uptake in axillary lymph node after COVID-19 vaccine was also confirmed in our study. Radiolabeled choline uptake in inflammation may be mediated by activation of cellular lines as monocytes and macrophages, expressing high synthesis of cellular membrane [13]. Some Authors already reported increased axillary lymph node or ipsilateral deltoid uptake with both [^18^F]Choline and [^11^C]Choline PET scans performed after mRNA vaccines [31,32].

Prostate cancer patients are still undergoing COVID-19 vaccination: the real prevalence of choline uptake in reactive axillary lymph nodes in this selected population needs to be addressed. Difference rate between 2-[^18^F]FDG and [^18^F]Choline reactive lymph nodes could be due to the low population examined with radiolabeled choline PET/CT, which could underestimate the real incidence of these findings in prostate cancer patients.

For the same reason, we did not observe similar findings with other considered PET tracers, mainly due to very low examined populations, a further limit of our study.

The real impact of tracer uptake in axillary lymph nodes after COVID-19 vaccine at PET/CT imaging with [^18^F]-6-FDOPA, [^68^Ga]Ga-DOTATOC [33], and [^18^F]-fluoride still needs to be addressed.

However, Eifer et al. [34] already reported the possibility of 2-[^18^F]FDG, [^68^Ga]Ga-DOTATATE, [^68^Ga]Ga-PSMA-11 and [^18^F]-6-FDOPA uptake in deltoid muscle and/or axillary lymph node in cancer patients submitted to COVID-19 vaccination and examined by PET/CT. Prudence should be recommendable when observing axillary, tracer-avid lymph nodes at PET/CT imaging with all cited tracers.

Presumably, [^18^F]-fluoride uptake should not be observed in reactive lymph nodes, due to the peculiar molecular pathway, particularly in absence of calcification.

## Conclusion

In the majority of patients submitted to COVID-19 vaccination, moderate 2-[^18^F]FDG uptake can be detected in the site of injection in deltoid muscle and/or in axillary lymph nodes, ipsilateral to vaccine administration. This can lead to a diagnostic conundrum in cancer patients with suspected neoplastic nodal involvement. Visual and semi-quantitative analysis with SUVmax of PET data can help in reaching the correct diagnosis, especially in patients with known secondary lymph node localizations of somatic cancers or active lymphomas.

Similar findings should be expected for radiolabeled choline and other PET tracers. Hybrid imaging with *low dose* CT and accurate patient anamnesis are sufficient to avoid misdiagnosis. In limited cases, ultrasonography can be included in the diagnostic work-up, improving confidence in correct identification of these false positive cases.

## Data Availability

The datasets used and/or analyzed during the current study are available to the corresponding author upon reasonable request.
